# Diminished transmission of drug resistant HIV-1 variants with reduced replication capacity in a human transmission model

**DOI:** 10.1186/s12977-014-0113-9

**Published:** 2014-12-14

**Authors:** Marieke Pingen, Ramin Sarrami-Forooshani, Annemarie MJ Wensing, Petra van Ham, Agata Drewniak, Charles AB Boucher, Teunis BH Geijtenbeek, Monique Nijhuis

**Affiliations:** Virology, Department of Medical Microbiology, University Medical Center Utrecht, Heidelberglaan 100, 3584CX Utrecht, the Netherlands; Department of Virology, Erasmus Medical Center, Rotterdam, the Netherlands; Department of Experimental Immunology, Academic Medical Center, University of Amsterdam, Meibergdreef 9, 1105AZ Amsterdam, the Netherlands

**Keywords:** HIV-1, Drug resistance, Transmission, Dendritic cells, Langerhans cells, M184V, K103N

## Abstract

**Background:**

Different patterns of drug resistance are observed in treated and therapy naïve HIV-1 infected populations. Especially the NRTI-related M184I/V variants, which are among the most frequently encountered mutations in treated patients, are underrepresented in the antiretroviral naïve population. M184I/V mutations are known to have a profound effect on viral replication and tend to revert over time in the new host. However it is debated whether a diminished transmission efficacy of HIV variants with a reduced replication capacity can also contribute to the observed discrepancy in genotypic patterns.

As dendritic cells (DCs) play a pivotal role in HIV-1 transmission, we used a model containing primary human Langerhans cells (LCs) and DCs to compare the transmission efficacy M184 variants (HIV-M184V/I/T) to HIV wild type (HIV-WT). As control, we used HIV harboring the NNRTI mutation K103N (HIV-K103N) which has a minor effect on replication and is found at a similar prevalence in treated and untreated individuals.

**Results:**

In comparison to HIV-WT, the HIV-M184 variants were less efficiently transmitted to CCR5^+^ Jurkat T cells by both LCs and DCs. The transmission rate of HIV-K103N was slightly reduced to HIV-WT in LCs and even higher than HIV-WT in DCs. Replication experiments in CCR5^+^ Jurkat T cells revealed no apparent differences in replication capacity between the mutant viruses and HIV-WT. However, viral replication in LCs and DCs was in concordance with the transmission results; replication by the HIV-M184 variants was lower than replication by HIV-WT, and the level of replication of HIV-K103N was intermediate for LCs and higher than HIV-WT for DCs.

**Conclusions:**

Our data demonstrate that drug resistant M184-variants display a reduced replication capacity in LCs and DCs which directly impairs their transmission efficacy. As such, diminished transmission efficacy may contribute to the lower prevalence of drug resistant variants in therapy naive individuals.

## Background

HIV variants harboring drug-resistance mutations are detected in approximately 10% of all newly diagnosed patients in the Western world [1,2]. Large clinical studies indicated that transmitted drug resistance may impact virological and immunological response to initial antiretroviral therapy [3,4]. A change from a methionine to valine or isoleucine at position 184 (M184V/I) in reverse transcriptase (RT) is the most frequently observed resistance mutation in patients experiencing treatment failure [5-7] but is only rarely observed in untreated, newly diagnosed individuals using population-based sequencing assays [1,2,5]. M184V/I causes high level resistance against the nucleoside reverse transcriptase inhibitors lamivudine and emtricitabine, but at the same time considerably decreases the processivity of reverse transcriptase (RT) resulting in a reduced viral replication capacity (RC) [8,9]. Contrary to M184V, the frequently observed RT mutation K103N has a similar prevalence in treated and untreated patients [5]. The presence of K103N causes high levels of resistance against the non-nucleoside reverse transcriptase inhibitors efavirenz and nevirapine. K103N has been described to have a modest effect on viral RC [10,11], and can persist for years after transmission to a new host [12].

The low prevalence of the M184V mutation in therapy-naive individuals may be explained by the reduced RC of this mutant, which can directly impair transmission efficacy and/or lead to reversion of M184V in the new host. When HIV variants harboring M184V are transmitted to a new host, rapid reversion of the M184V variant has been documented (reviewed in [12]). Accordingly, the M184V variant can be detected as a minority species in recently infected individuals using very sensitive assays, which is suggestive of reversion to wild type [5]. In addition, diminished transmission efficacy of the M184V variant has been suggested based on mathematical modeling and a macaque SHIV model [13,14]. However, the impact of RC on transmission efficacy has never been investigated in a human transmission model.

Sexual HIV transmission is an inefficient process during which a limited number of virions initiate an infection in a new host, resulting in a severe transmission bottleneck [15,16]. Although CD4^+^ T cells are the predominant target cells of HIV, it has been postulated that dendritic cells (DCs) naturally residing in the genital mucosa play a major role during sexual transmission [17-20]. Within the genital mucosa, Langerhans cells (LCs) reside in the epithelial layer and are the first DC subset encountered by HIV. LCs express the C-type lectin receptor langerin that captures HIV, leading to internalization and degradation of HIV. LCs therefore function as a natural barrier against HIV transmission. However, when the protective function of langerin is saturated, for example in the presence of a high inoculum or when langerin is downregulated due to cell maturation, LCs can become infected and subsequently transmit HIV to T cells [21]. Furthermore, DC-SIGN^+^ DCs, which reside in the sub-epithelium, can transmit HIV to T cells. These DCs express the C-type lectin DC-SIGN that efficiently captures HIV and transmits the virus to T cells [22]. Transmission of HIV by DCs and LCs may occur either as a result of infection of DCs/LCs and subsequent *de novo* virus replication (in *cis*), or by uptake and transfer of virions (in *trans*). Both mechanisms have been observed in *in vitro* studies [23-25]. The objective of this study was to investigate the transmission efficacy of the HIV-1 M184V/I RT variants. We used an HIV transmission model containing primary human DCs to compare the transmission efficacy of HIV harboring M184V/I to wild type HIV. With this virus panel, we demonstrated that the M184V/I variants were less efficiently transmitted to CCR5^+^ Jurkat T cells by both LCs and DCs, which was due to the lower RC of the M184V/I variants in both DC subsets. These results clearly imply a role for HIV RC in transmission efficacy and provide an additional explanation for the low prevalence of HIV M184V/I in therapy naïve individuals.

## Results

### Impact of drug resistance mutations on transmission by LCs and DCs

We hypothesized that due to a diminished replication potential [9], HIV harboring M184V/I is less efficiently transmitted than HIV-WT or drug resistant virus variants with a high RC [11]. Therefore, we compared the transmission efficacy of HIV-M184V/I to HIV-WT and HIV-K103N. To gain more insight in the impact of RC on transmission efficacy, we additionally investigated the drug resistant HIV-M184T variant. The RC of HIV-M184T is even more impaired than the RC of M184V/I, and as such this variant is rarely observed *in vivo* but can be selected *in vitro* [9,26]. Since DCs play an important role during HIV transmission [17,19], we assessed the transmission efficacy of this virus panel by two subsets of DCs: primary human LCs and human monocyte-derived immature DCs. Migratory LCs were isolated from the epidermis of skin obtained after plastic surgery from multiple healthy donors (purity: >95%, described in ref [21]) and exposed to HIV for four days to enable infection. A low and a high inoculum (17.5 and 100 ng p24) were used in the infections to ensure that the observed results are due to differences in infection and replication rather than caused by differences in titre of the virus stock or saturation of our transmission model. Virus input was based on p24 protein levels as this excludes any impact of the RC of the virus on viral input.

**Figure 1:**
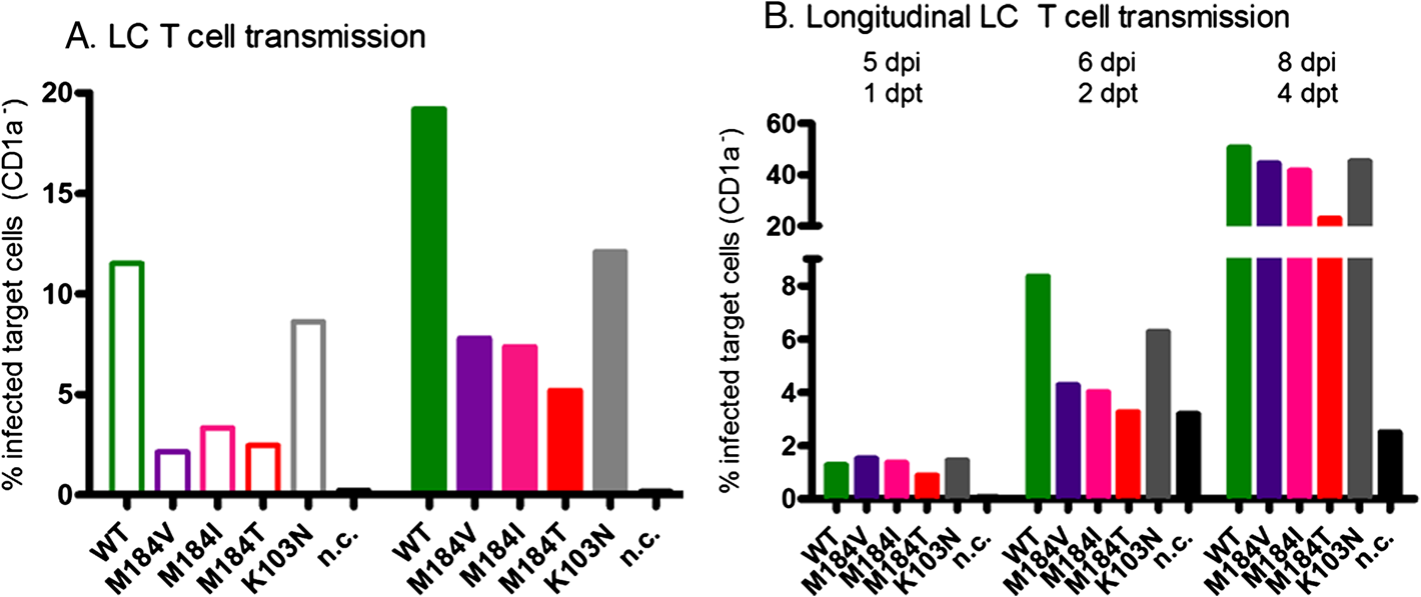
**Diminished transmission of M184 variants by LCs.** LCs were exposed to the equivalent of 17.5 (open bars) or 100 ng (closed bars) p24 for up to four days, extensively washed and co-cultured for four days with CCR5^+^ Jurkat T cells. Cells were stained with antibodies against CD1a as a marker for LCs and intracellular p24 for HIV infection and analyzed by flow cytometry. **A**: The percentage of infected target CCR5^+^ Jurkat T cells (CD1a negative cells) at 2 days post transmission (dpt) (6 days post infection; dpi) (n = 3, representative donor depicted). **B**: The percentage of infected target cells (CD1a negative cells) at 1, 2 and 4 days post transmission (1 donor, only 100 ng p24 infection is shown). Abbreviation: n.c.: no infection control.

To model HIV transmission, the HIV exposed LCs were extensively washed and subsequently co-cultured with CCR5^+^ Jurkat T cells for two days. Infection of these target T cells was determined by flow cytometry using intracellular p24 staining for HIV infection. HIV-M184V, −I and –T (combined: HIV-M184 variants) were less efficiently transmitted by LCs to T cells than the HIV-WT, whereas the level of transmission of HIV-K103N was intermediate (n=3 donors, Figure 1A). To verify that these results are not due to saturation of the target cells, we longitudinally assessed the transmission efficacy of LCs to target cells. For up to four days post transmission, similar transmission kinetics were observed (Figure 1B).

**Figure 2:**
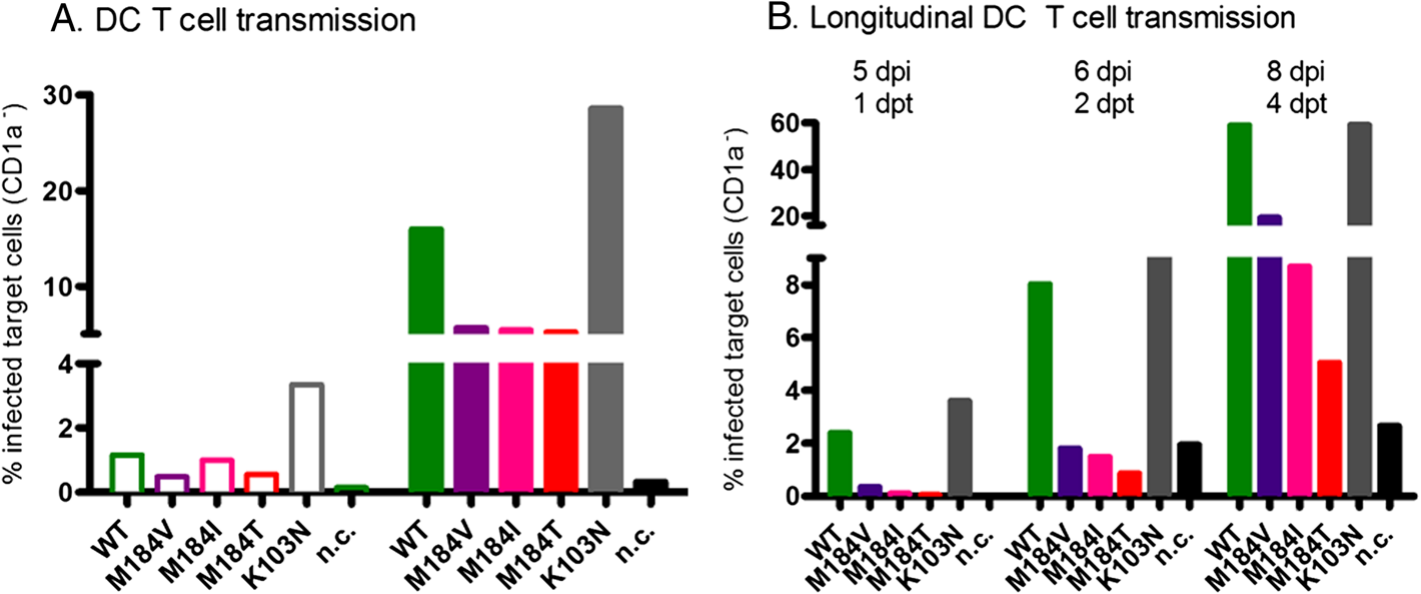
**Diminished transmission of M184 variants by DCs.** DCs were exposed to the equivalent of 17.5 (open bars) or 100 ng (closed bars) p24 for four days, extensively washed and co-cultured for two days with CCR5^+^ Jurkat T cells. Cells were stained with antibodies against CD1a as marker for DCs and intracellular p24 for HIV infection and analyzed by flow cytometry. **A**: The percentage of infected target cells (CD1a negative cells) at 2 days post transmission (dpt) (6 days post infection; dpi) (n = 4, representative for 3/4 donors). **B**: The percentage of infected target cells (CD1a negative cells) at 1, 2 and 4 days post transmission (n = 2, 100 ng p24 infection is shown). Abbreviation: n.c.: no infection control.

Monocyte-derived immature DCs were used as a model for sub-epithelial DCs [27]. Transmission by DCs obtained from multiple donors (purity: >90% described in ref [27]) was investigated in the aforementioned transmission model. In accordance with the results obtained by LCs, transmission of HIV-M184 variants from DCs to target cells was lower than HIV-WT for 3/4 donors. The transmission rate of HIV-K103N was even higher than HIV-WT in all but one experiment (Figure 2A). Again, longitudinal transmission experiments demonstrated similar transmission kinetics up to 4 days post transmission (Figure 2B).

### Replication capacity of mutant viruses in target cells

**Figure 3:**
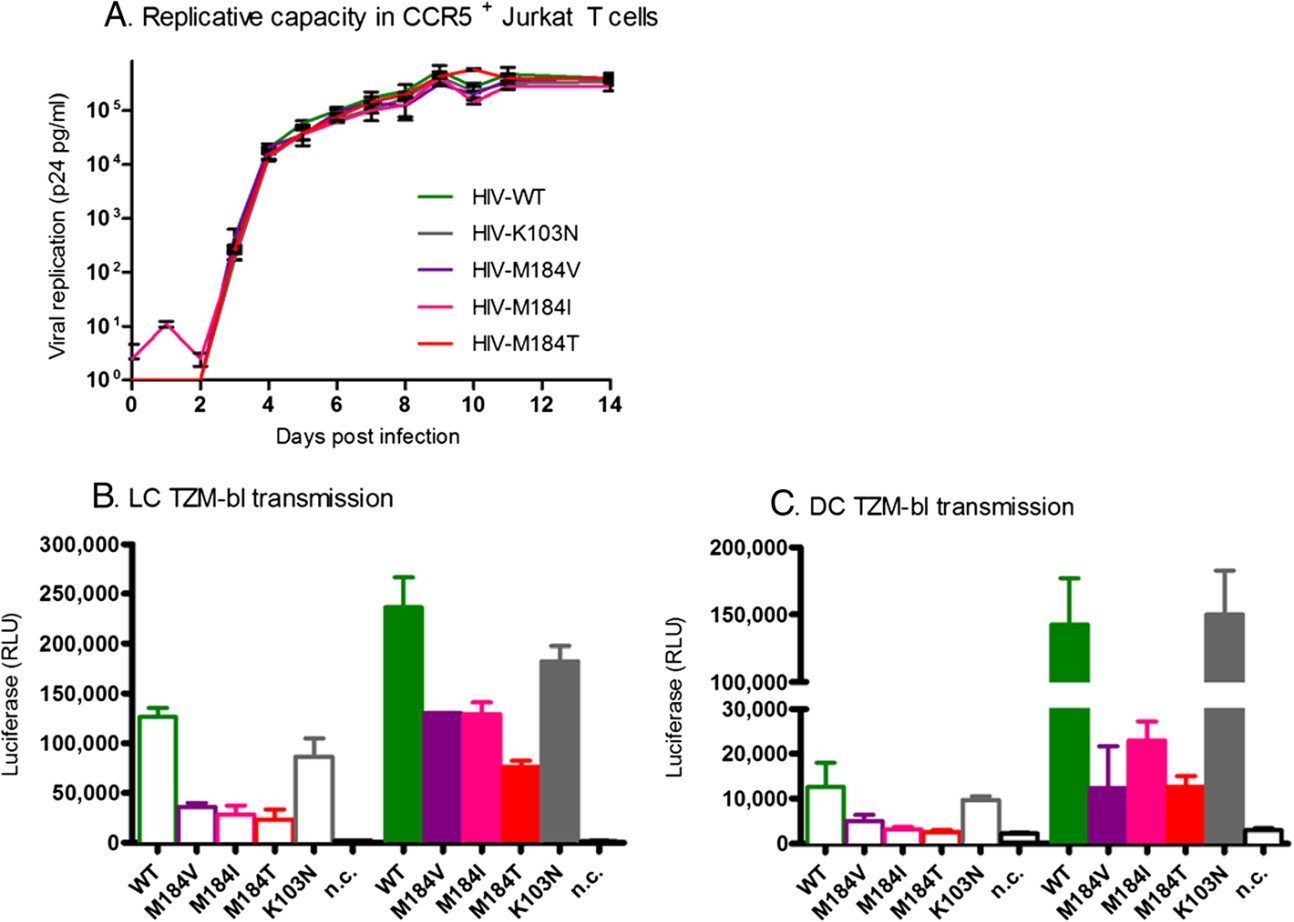
**The diminished transmission of M184 variants is not caused by replication in target cells. A**: To determine the replication capacity of the virus panel in target cells, CCR5^+^ Jurkat T cells were infected in the absence of drugs and p24 production was monitored daily. Average infection with standard deviation is depicted. **B**-**C**: LCs **(B)** or DCs **(C)** were exposed to the equivalent of 17.5 (open bars) or 100 ng (closed bars) p24 for four days, extensively washed and co-cultured for two days with TZM-bl cells pre-incubated with indinavir. Infection was measured by luminescence compared to HIV-WT. Data are representative for the average with SD for 1 (LCs) and 2 (DCs) donors in duplo. Abbreviations: WT: HIV-WT, M184V: HIV-M184V, M184I: HIV-M184I, M184T: HIV-M184T, K103N: HIV-K103N, n.c.: no infection control.

Although the RC of HIV-M184V and HIV-M184I is decreased in primary T cells, we have previously shown that the RC of the mutants is similar to HIV-WT in a T cell line [9]. To confirm that the observed differences in transmission efficacy are caused by transmission from DCs rather than replication in the target cells, we assessed the RC of all viruses in the CCR5^+^ Jurkat T cells that were used in the transmission experiments. No apparent differences in RC were observed between the mutant and wild type viruses (Figure 3A).

As an additional control, the transmission experiments were repeated using different target cells and a single cycle read out. Therefore, LCs and DCs were exposed to the HIV variants as aforementioned, but replication in TZM-bl target cells was limited to one round of replication by (pre-)incubation with the protease inhibitor indinavir. In line with the results obtained with CCR5^+^ Jurkat T cells, HIV-M184 variants and HIV-K103N were less efficiently transmitted by LCs than HIV-WT. The transmission efficacy of LCs and DCs to TZM-bl target cells was also comparable to the CCR5^+^ Jurkat T cells; the HIV-M184 variants were less frequently transmitted as HIV-WT and the HIV-K103N transmission efficacy was comparable to HIV-WT (Figure 3B-C). These data suggest that the diminished transmission of M184 variants by LCs and DCs is not caused by differential RC in the target cells.

### Replication capacity of mutant viruses in LCs and DCs

**Figure 4:**
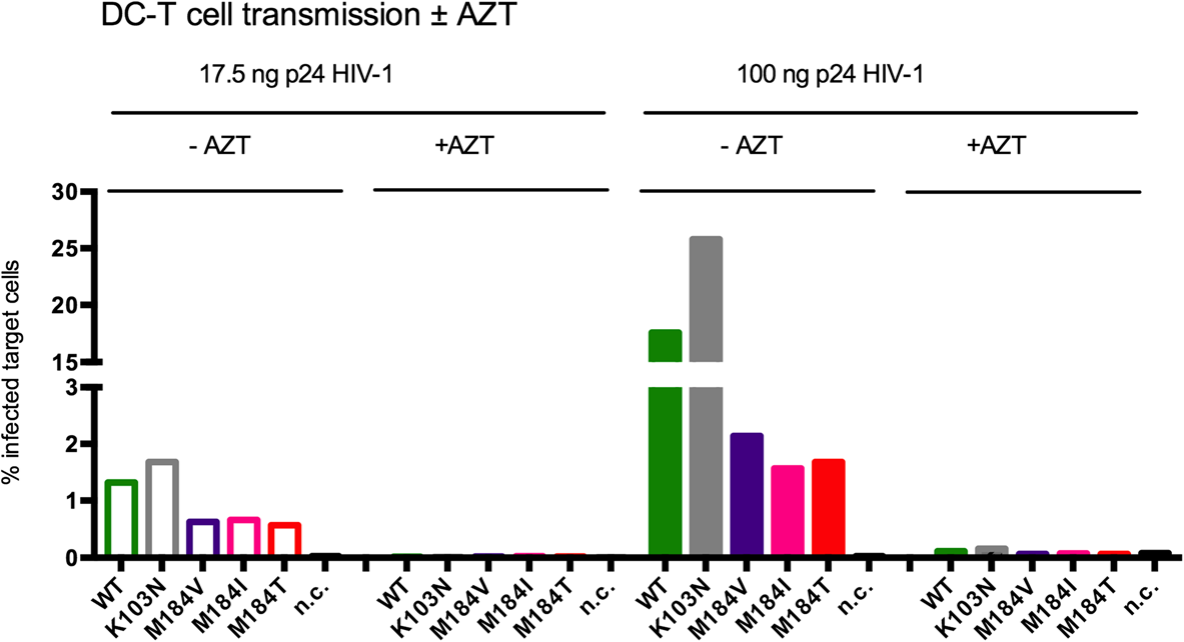
**Transmission to target cells is driven by** ***cis*** **infection of DCs.** DCs were infected in the absence and presence (diagonal striping marks) of AZT, a potent inhibitor of RT. All infections were started with virus equivalent to 17.5 ng (open bars) and 100 ng (filled bars) p24. Four days post infection, cells were extensively washed and co-cultured for two days with CCR5^+^ Jurkat T cells. Cells were stained with antibodies against CD1a as marker of DCs and intracellular p24 for HIV infection and analyzed by flow cytometry. The percentage p24 positive target cells is depicted. Data are representative for 2 donors.

We have recently demonstrated that transmission by LCs largely occurs *in cis*, by virus replication [25]. To investigate the impact of active replication of HIV-1 in DCs on *in vitro* HIV-1 transmission, DCs were infected in the presence of AZT to inhibit replication before co-culture with CCR5^+^ Jurkat T cells. As such, only *trans* infection by DCs to T cells was allowed. Indeed, inhibiting viral replication in DCs efficiently abrogated transmission to CCR5^+^ Jurkat T cells, demonstrating a major role for infection by DCs in viral transmission (Figure 4).

**Figure 5:**
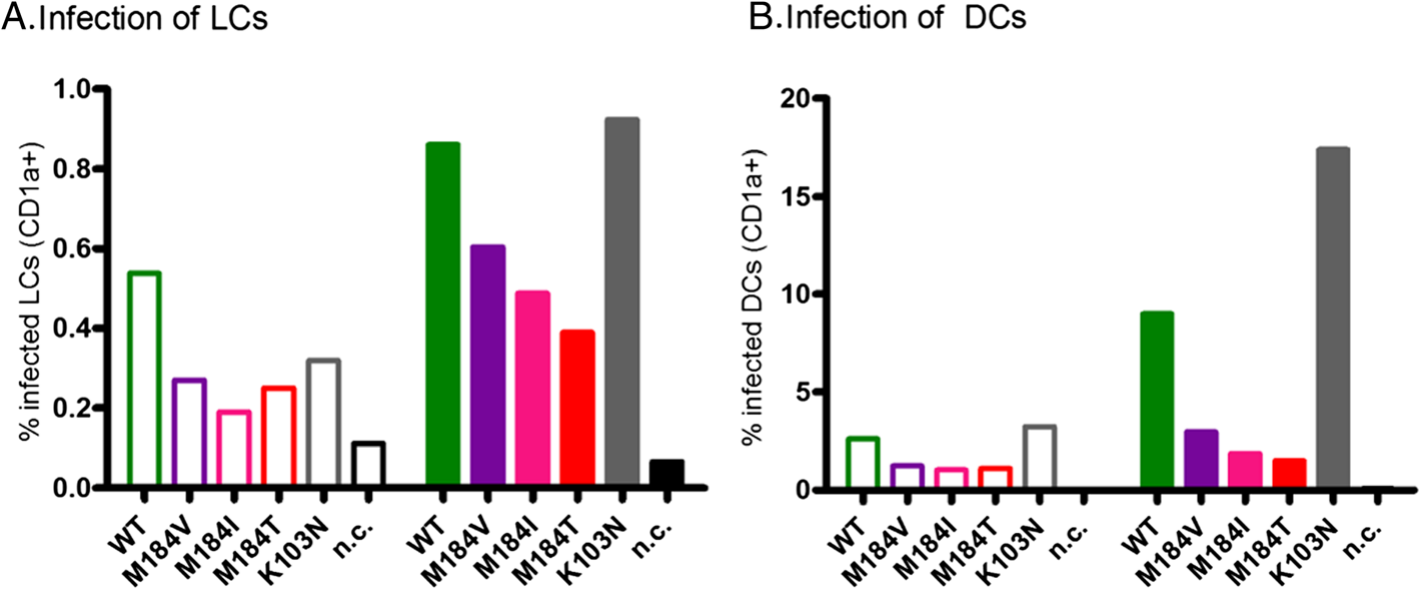
**Lower infection of LCs and DCs by M184 variants.** HIV infection of LCs **(A)** or DCs **(B)** by a panel of HIV-1 drug resistance variants (M184V, −I, −T and K103N) was measured after six days. All infections were started with virus equivalent to 17.5 ng (open bars) and 100 ng (filled bars) p24. Cells were stained with antibodies against CD1a as marker of LCs and intracellular p24 for HIV infection and analyzed by flow cytometry. The percentage p24 positive LCs **(A)** or DCs **(B)** is depicted. Abbreviation: n.c.: no infection control.

Next, we investigated if the observed differences in transmission efficacy can be explained by the RC of HIV-WT and the drug resistant viruses in LCs and DCs. To do so, LCs or DCs were exposed to all viruses for six days to enable infection [23], after which the infection rate of CD1a positive cells (marker for LCs/DCs) was measured by detection of intracellular p24 by flow cytometry. It was previously described that LCs have a low susceptibility to HIV infection [21]. Although the percentage of infected LCs was indeed low, the level of infection by HIV-M184 variants was clearly reduced as compared to infection by HIV-WT. In agreement with the LC transmission experiments, the infection level of LCs by HIV-K03N was intermediate (Figure 5). Furthermore, the level of infection of DCs was also in line with the observed transmission efficacy of DCs to T cells. Compared to HIV-WT, the infection rate of HIV-M184 variants was lower in DCs. The infection rate of HIV-K103N was higher than to HIV-WT, which is in agreement with the observed transmission data (Figure 5).

**Figure 6:**
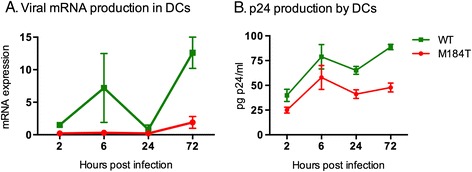
**Detailed analysis of replication capacity of HIV-M184T in DCs.** **A**-**B**: DCs were infected with 17.5 ng p24 HIV-WT or HIV-M184T. Intracellular viral mRNA transcription was determined using RT-qPCR relative to expression of the housekeeping gene glyceraldehydes phosphate dehydrogenase (GAPDH) **(A)** and viral production was measured by ELISA for p24 in the supernatant **(B)**. Average ± SD of DCs from 3 donors infected in duplo is shown.

In addition, we investigated the level of intracellular mRNA production as a measure of successful completion of the RT process and integration of the proviral DNA. RT-qPCR on intracellular RNA demonstrated that HIV-WT was able to transcribe a higher amount of the viral mRNA Tat-Rev (3 donors, Figure 6A) than HIV-M184T. Furthermore, HIV-WT was also able to produce more virus than HIV-M184T as determined by HIV-CA (p24) ELISA of in the supernatant (3 donors, Figure 6B).

**Figure 7:**
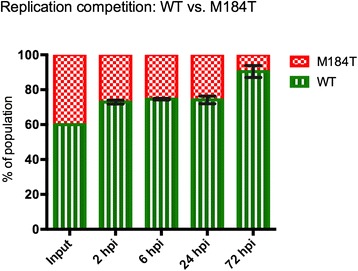
**Replication competition experiments of WT vs. M184T.** At 2, 6, 24 and 72 hours post infection (hpi), the relative presence of HIV-WT (green, horizontal stripes) and HIV-M184T (red, checkered) were determined by population sequencing. Mean ± SEM of experiments using three different donors in duplicate is depicted.

Finally, we investigated the relative viral RC in DCs. DCs from three different donors were infected with a mixture of HIV-WT and HIV-M184T, and their relative replication capacity was determined by analysis of their frequency in the viral population over time. It was shown that HIV-WT rapidly outcompeted HIV-M184T, confirming the low RC of HIV-M184T in DCs (3 donors, Figure 7). Combined, these data strongly indicate that the observed diminished transmission efficacy of HIV-M184 variants is caused by decreased RC in DCs.

## Discussion

We have investigated the impact of drug resistance mutations in HIV RT on viral transmission efficacy. K103N and M184V are both frequently observed in patients experiencing therapy failure, but whereas M184V is rarely detected in newly diagnosed patients, K103N is often observed [2,7]. It was debated whether a diminished transmission efficacy could contribute to this observed discrepancy in prevalence. We compared transmission of wild type HIV with HIV-M184V in an HIV transmission model containing primary human LCs or DCs. In addition, we investigated transmission of HIV-K103N and HIV-M184T as controls. We demonstrated that transmission by LCs and DCs to T cells is affected by the replication capacity defect caused by the M184 mutation.

Our results are in line with a study that compared transmission of SHIV wild type and M184V in rhesus macaques. In a repeated low-dose rectal transmission model, a larger inoculum was needed to successfully infect macaques with a SHIV variant containing M184V, indicating that mucosal transmissibility of the M184V variant is lower than wild type [13].

It has been known for a long time that the RC of HIV harboring M184V/I/T is reduced in primary T cells [9,26]. Our data demonstrate that replication in primary LCs and DCs is also affected and as a result, transmission to T cells is diminished. We have previously demonstrated that the impact of M184VIT is more pronounced in primary cells containing low levels of dNTPs [9]. In myeloid cells such as DCs, SAMHD-1 lowers the intracellular dNTP levels [28] and as such may impair the replication of the HIV-M184 variants [9].

The frequently observed NNRTI-related mutation K103N has been described as having a modest impact on RC by several [10,11,29], but not all [30] previous studies. This discrepancy may be caused by differences in the assays that were used to determine viral RC in these studies, such as the viral genetic background or cell type. In our HIV transmission model, the infection of and transmission by primary DCs and LCs of HIV-K103N was consistently higher than the HIV-M184 variants. Remarkably, the level of infection of DCs by the K103N mutant was even higher than HIV-WT in the majority of donors.

Several studies have addressed transmission efficacy in humans by comparing the prevalence of drug-resistant HIV variants in newly diagnosed patients and treatment-experienced patients [14,31,32]. These studies observed a reduced transmission rate of HIV variants harboring drug resistance mutations. Such *in vivo* approaches measure the net result of potential differences in transmission efficacy combined with potential reversion of drug-resistance mutations after transmission to the new host. M184V is known to revert rapidly after transmission [12]. Indeed, the Swiss HIV cohort study described a lower prevalence of HIV-M184V in acutely infected HIV individuals compared to chronically infected patients [33]. Using *in vitro* experiments, we were able to exclusively investigate the impact of drug resistance mutations on the transmission efficacy. DCs can either be productively infected (*cis*-infection), or transfer captured virions by *trans* infection [34-36]. In our *in vitro* HIV transmission model, *cis* infection of LCs and DCs plays an important role [21,37]. Using this *in vitro* model, we were able to demonstrated that HIV-M184 variants not only have a lower RC in primary T cells, but also in DCs and LCs which decreases the transmission efficacy of these drug resistant HIV variants.

Our data indicate that the RC of HIV variants with RT drug resistance mutations can impact the transmission efficacy. This may contribute to the discrepancy of the prevalence of M184V in treatment-experienced and naive individuals. In addition to RT drug resistance mutations, also variants harboring protease or integrase inhibitor resistance mutations decrease viral RC [38,39]. Determination of the impact of mutations affecting other steps in the viral replication cycle on transmission efficacy may enhance our understanding of the role of RC in transmission. In addition, DCs are important targets for the transmission of several other viruses such as measles virus, herpes simplex virus and phleboviruses [40-42]. As such, one could hypothesize that differences in RC in DCs also impact transmission of other viruses.

## Conclusions

We have shown a diminished transmission of M184 variants from LCs and DCs to target cells, which was likely caused by the lower RC of M184 variants in LCs and DCs. Therefore, a diminished transmission efficacy of drug-resistant variants provides an additional mechanism explaining the observed discrepancy in prevalence of replication-deficient drug-resistant HIV variants in treatment-experienced and naive individuals.

## Methods

### Virus panel

The site-directed mutants M184V, M184I and M184T were previously generated [9,26] in the background of HXB2. The mutation resulting in amino acid change K103N was introduced in HXB2 by site-directed mutagenesis using the previously described vector system with addition of primer K103N (5′-GTTACTGATTTGTTCTTTTTTAACCC-′3) [43]. Tropism of all viral variants was changed from CXCR4-tropic to CCR5-tropic by replacing the HXB2-V3 loop with the V3 loop of the CCR5-tropic lab strain BaL). HXB2-cBaL, referred to as HIV-WT, was generated by introducing a unique *BmgBI*-restriction site at position 7091 in HXB2. After restriction with BmgBI and NheI, nucleotides 7091 to 7260 of HXB2 were replaced by V3 of BaL. Subsequently, the plasmids containing drug-resistance mutations were restricted by NcoI and NheI and nucleotides 5675 to 7260 of these plasmids were replaced by the corresponding region of HXB2-cBaL.

Virus was obtained by transfection of HEK293T cells with plasmid DNA using Lipofectamine 2000 (Invitrogen) according to the manufacturer’s protocol. To exclude a possible influence of different batches, all steps of virus production were performed synchronized. Virus was quantified by p24 analysis.

### Cells

CCR5^+^ Jurkat T cells were generated and maintained as previously described [21]. TZM-bl cells that express CCR5 were obtained through the NIH AIDS Research and Reference Reagent Program and maintained as recommended. Donor PBMCs were obtained by Ficoll-Paque density gradient centrifugation of heparinized blood from five HIV-seronegative donors, pooled and stored at −80°C until use. PBMCs were stimulated for three days with phytohaemagglutinin (2 mg/l) in culture medium (RPMI 1640 with L-glutamine (Lonza, Verviers, Belgium), 10% fetal bovine serum (Sigma-Aldrich, Zwijndrecht, the Netherlands) and 10 μg/ml gentamicin (Invitrogen, Breda, the Netherlands). LCs and DCs were obtained from multiple donors as previously described [21]. In short, for LC isolation, epidermis was separated by dispase II treatment (1 mg/ml, Roche Diagnostic Systems, Somervolle, NJ) and cultured until LC maturation and migration. The resulting cell suspension was purified by CD1a positive selection using MACS, according to the manufacturer’s protocol (Miltenyi Biotec, Auburn, CA). This procedure yielded a >95% pure CD1a^+^ LC population (Figure 1A). The small population of contaminating cells mostly consists of keratinocytes. Monocytes were isolated by density centrifugation of PBMCs and cultured for five days in the presence of 800 U/ml IL-4 and 1000 U/ml GM-CSF to stimulate differentiation into DCs. The purity of obtained DCs was >90% (Figure 2A). Less than 5% of the contaminating cells are T cells which are not found to be productively infected, and excluded from analysis using gating on CD1^+^ cells in flow cytometry.

### Viral replication capacity

RC in CCR5^+^ Jurkat T cells was determined in duplicate by infecting 2×10^6^ cells with the equivalent of 40 ng p24 of each virus. After two hours of inoculation, cells were washed twice with RPMI 1640 medium with L-glutamine and resuspended in 10 ml culture medium with 5 U/ml IL-2 (Roche, Mannheim, Germany). 300 μl cell-free supernatant was harvested daily for p24 analysis. Cultures were maintained for 14 days.

Infection of DCs and LCs was determined by infecting 50,000 cells with 17.5 or 100 ng p24. After 6 days of infection, cells were stained with α-CD1a as a marker for DCs and α-p24 as marker for productive HIV infection. Living cells were gated based on forward and sideward scatter; DCs were distinguished from contaminating cells based on CD1a expression.

In replication competition experiments, DCs were infected with 100 ng p24 of both HIV-WT and HIV-M184T. After inoculation, cells were washed and the ratio of mutant and WT viral RNA was determined using viral population sequencing.

Intracellular mRNA was isolated using the mRNA capture kit according to manufacturer’s protocol (Roche). cDNA was synthesized using a RNA-to-cDNA kit (Promega). Quantitative PCR was performed to determine Tat-Rev, an early expressed viral mRNA, using a SYBR green approach using the following primers: 5′-ATGGCAGGAAGAAGCGGAG-3′ and 5′-ATTCCTTCGGGCCTGTCG-3′. Viral gene expression was normalized to housekeeping gene GAPDH as previously described [44].

### HIV transmission

50,000 DCs or LCs were infected with the equivalent of 17.5 and 100 ng p24 of all HIV variants for four to five days. After extensive washing, DCs or LCs were added to the target cells, which were either 50,000 CCR5^+^ Jurkat T cells or TZM-bl cells that were pre-seeded on 96 well plate (confluence 70%). After 1–4 days of co-culture, infection was measured by flow cytometry as described above (CCR5^+^ Jurkat T cells) or by luminescence (TZM-bl cells). TZM-bl cells were (pre-)incubated with 1,000 nM indinavir to investigate a single replication cycle after transmission.

### Antibodies

The following antibodies were used: CD1a-FITC (BD Pharmingen, San Diego, CA, USA), CD1a-APC, CD3-PE (both BD Bioscience, San Jose, CA, USA), CD4-PerCP (BD Pharmingen), CD4-Alexa488 (Biolegend, San Diego, CA, USA), CCR5-APC (CD195) (BD Pharmingen), CXCR4-PerCP (R&D systems, Minneapolis, MN, USA), Langerin-PE (CD207), p24-PE (both Beckman Coulter, Fullerton, CA, USA).
